# Efficient Removal of Cr(VI) Ions in Petrochemical Wastewater Using Fe_3_O_4_@*Saccharomyces cerevisiae* Magnetic Nanocomposite

**DOI:** 10.3390/nano12183250

**Published:** 2022-09-19

**Authors:** Wei Long, Zhilong Chen, Jie Shi, Shilin Yang

**Affiliations:** 1 Guangdong Provincial Key Laboratory of Petrochemical Pollution Process and Control, Guangdong University of Petrochemical Technology, Maoming 525000, China; 2 College of Chemistry, Guangdong University of Petrochemical Technology, Maoming 525000, China

**Keywords:** Fe_3_O_4_@*SC*, petrochemical wastewater, Cr(VI) ions, adsorption, magnetic nanocomposite

## Abstract

*Saccharomyces cerevisiae* (*SC*) is a widely available biobased source for function material. In this work, a kind of new efficient magnetic composite adsorbent containing Fe_3_O_4_ and *SC* was prepared successfully and used for the removal of Cr(VI) ions in petrochemical wastewater. The morphology and structure of this magnetic adsorbent were characterized with FT-IR, TG, XRD, VSM, SEM and XPS. The effect of the different factors such as pH, adsorption time, initial Cr(VI) ions concentration and adsorption temperature on the adsorption behavior were investigated. The results showed that 10%-Fe_3_O_4_@*SC* exhibited high removal rate, reutilization and large removal capacity. The corresponding removal capacity and removal rate could reach 128.03 mg/g and 96.02% when the pH value was 2, adsorption time was 180 min, and initial Cr(VI) ions concentration were 80 mg/L at 298 K. The kinetics followed the pseudo-first-order, which indicated that the adsorption behavior of 10%-Fe_3_O_4_@*SC* for Cr(VI) ions belonged to the physical adsorption and chemical adsorption co-existence. The thermodynamic study showed that the adsorption process was spontaneous and exothermic. It still showed better adsorption performance and reutilization after the fifth adsorption-desorption experiment. The possible mechanism of Cr(VI) ions adsorption onto the 10%-Fe_3_O_4_@*SC* magnetic adsorbent has been discussed. Hence, this new adsorbent will be a candidate for industry-level applications in petrochemical wastewater containing Cr(VI) ions.

## 1. Introduction

With the rapid growth in technology and industries, heavy metals pollution in the environment is more and more serious, which can comminate human health [1]. Particularly, petrochemical wastewater is so complex and there are so many chemistry components that the treatment process of petrochemical wastewater is so difficult and cannot be applied smoothly in different regions [2]. There are thousands of kinds of aromatic hydrocarbons, acids, esthers, phenols or alcohols in the petrochemical wastewater, and much organic substance can be congregated and burned easily, but there are some soluble organic substance always in the water solution [3]. Further cleaner treatment maybe depend on the adsorption behavior of plants. Heavy metals, including cadmium, lead, chromium, mercury, zinc and copper ions, have been widely dispersed into the petrochemical wastewater [4]. Heavy metals are not biodegradable and easily accumulate in living organisms, resulting in elevated heavy metal concentrations in the environment or animal [5]. Thus, the purification process of heavy metal ions is essential, which is equally as important as the treatment of organic substance [6].

Azimi et al. [7] summarized various types of techniques for removing heavy metal ions in the industrial wastewater solution, e.g., ion-exchange, membrane process, osmosis, and electrolytic technologies. However, many techniques are costly and may inevitably be accompanied by the generation of by-products [8]. Some techniques, including extraction, are very dangerous because of the use of a flammable organic phase [9]. Especially, precipitation reaction may be the ideal process as it is ascribed to many metal hydroxides that can be transformed as precipitate in the proper alkaline solution. This process needs much sodium hydroxide or ammonia, the cost is not cheap, and the corrosiveness is also a serious threat to the environment [10]. Adsorption is a good method for the removal of metal ions as it means the removal of heavy metal ions via their adsorption onto adsorbents, and it has been considered the most versatile, as a relatively simple and cost-effective technique [11]. The concentrations of heavy metal ions in water solution are reduced through their adsorption onto the surfaces of adsorbent materials, and the metal ions can eventually be removed from the water solution by removing the adsorbents [12]. Hence, adsorbence is a very important role for the removal of heavy metal ions.

Typically, modified activated carbon is one of the most adopted adsorbent materials due to its high efficiency in heavy metal removal, which is ascribed to functional groups on the large surface area and abundant micro-pores, so the heavy metals could be adsorbed on the surface of adsorbent [13]. The high cost of generating activated carbon from source materials, such as lignite, coal and wood, attracts many researchers to finding new cheap adsorbent [14]. Clay minerals, oyster shells, and certain waste products from industrial facilities can be considered to prepare the new functional adsorbent [15]. In addition, Demirbas [16] tested the applicability of by-products from the agricultural industry, including barks, hull, fruit peel and tea waste as the adsorbents of heavy metal ions in contaminated water, but these adsorbents had not been used in petrochemical wastewater [17].

*Saccharomyces cerevisiae* is one of the largest industrial products. It also is one of the cheapest by-products of beer winery [18]. The applicability of *Saccharomyces cerevisiae* for metal ions biosorption is attributed to the ease of its cultivation on a large scale, safety, high biomass production, use of cheap media, and high biosorption capacity [19]. *Saccharomyces cerevisiae* biomass is obtained easily and its use remains largely unexploited, so its disposal is frequently for solving environmental engineering problem, such as wastewater treatment [20]. The research of *Saccharomyces cerevisiae* started many years ago, when Bennetzen et al. [21] determined the DNA sequence of the *Saccharomyces cerevisiae* gene for the fermentative yeast alcohol dehydrogenase. Zinicovscaia et al. [22] had used microalga Spirulina platensis for rhenium removal from a single-component batch system and two rhenium-containing industrial effluents. They also used *Saccharomyces cerevisiae* biomass for the adsorption of rhenium, copper and molybdenum ions [23]. Maximum removal of rhenium (75–84%) and molybdenum (85%) were obtained at pH 2.0; Langmuir isotherm showed the maximum yeast adsorption capacities toward rhenium ions ranged between 7.7 and 33 mg/g. Rossi et al. [24] used *Saccharomyces cerevisiae* biomass as a biosorbent for the adsorption of Pb^2+^, Cr^6+^ and Cd^2+^ in water. The removal affinity occurred in the following order: Pb^2+^ > Cr^6+^ > Cd^2+^, with maximum monolayer removal capabilities estimated by the Langmuir model of 179.1, 72.1 and 30.7 mg/g, respectively. Hence, as an efficient bioadsorbent, *Saccharomyces cerevisiae* has potential advantages for the removal of heavy metal ions.

The content of Cr(VI) ions in petrochemical wastewater is not ignored, and the adsorption of Cr(VI) ions is more difficult than other heavy metal ions [25]. Rice husk-based activated carbon was used for the adsorption of Cr(VI) ions in water. The carbons prepared in different activation conditions have different adsorptions due to their different pore size or pore volume, and the adsorption increased with the change in trend of pore size or pore volume [14]. The spent activated clay was investigated for the adsorption of Cr(VI) ions in wastewater, and the maximum adsorption capacities for Cr(VI) ions ranged from 0.743 to 1.422 mg/g with the temperature from 4 to 40 °C under a condition of pH 2.0. The process of removal may be spontaneous at a high temperature and endothermic smoothness [26]. Mesoporous silica materials had also been tested for adsorption of Cr(VI) ions in water; the channels of as-synthesized mesoporous silica are essentially grafted with abundant amino groups and loaded with Cr(VI) ions and the maximum Cr(VI) ions loading at 35 °C reached 2.86 mmol/g [27]. Kinetics and thermodynamic studies were performed in the adsorption Cr(VI) ions from aqueous solution by adsorbent prepared from paper mill sludge [28]. In all, many bioadsorbents were suitable for the adsorption of Cr(VI) ions in water, but the adsorption performance is not ideal.

Thus, this study aimed to develop adsorbent using the residual *Saccharomyces cerevisiae* from the brewing industry, purification and design magnetic nanocomposite material, which can be used to evaluate the adsorbent potential of Cr(VI) ions in petrochemical wastewater. The Fe_3_O_4_@*Saccharomyces cerevisiae* nano magnetic adsorbent material was prepared and characterized (Fe_3_O_4_@*SC*). The optimum adsorption pHs were detected, and the adsorption equilibrium was analyzed using isothermal models. Adsorption tests were performed in the diluted petrochemical wastewater.

## 2. Materials and Methods

### 2.1. Chemicals and Instruments

Beer yeast sludge (containing *Saccharomyces cerevisiae*) was collected after the fermentation process in Wuchuan Yanjing brewery and further purification or modification, were finished in the laboratory. Real petrochemical wastewater samples were collected from the discharged sewage of Maoming Petrochemical Refining Co., Ltd (Guangdong, China). The other chemicals in this study were consumed as provided by suppliers without any further modifications. Formaldehyde, anhydrous ethanol, polyethylene glycol (molar mass: 600), K_2_Cr_2_O_7_, 1,5-diphenyl carbazide, FeCl_3_·6H_2_O and FeCl_2_·4H_2_O were purchased from Shanghai McLean Biochemical Technology Co., Ltd. (Shanghai, China). Hydrochloric acid, sulfuric acid, sodium hydroxide and ammonia water were provided by Tianjin Damao Chemical Reagent Co., Ltd. (Tianjin, China).

### 2.2. Preparation of Fe_3_O_4_@SC

Beer yeast sludge containing lots of *Saccharomyces cerevisiae* was chosen for treatment. Firstly, 100.00 g beer yeast sludge was subjected to six washes with 500 mL distilled water and anhydrous ethanol, followed by centrifugation at 6000 rpm for 15 min in order to remove the soluble substance. Secondly, vapor sterilization was performed to remove microorganisms in the beer yeast powder, and the powder also was washed by 300 mL distilled water and soaked in 100 mL formaldehyde solution (37 wt%) for 2 h. The next step, the powder was washed by 500 mL distilled water and soaked in 200 mL HCl solution (0.1 mol/L) to activate for 3 h, the brown *Saccharomyces cerevisiae* was separated from beer yeast powder by centrifugation method and washed by 300 mL distilled water and 300 mL anhydrous ethanol again. Then, the brown *Saccharomyces cerevisiae* was vacuum dried carefully at 60 °C for 12 h and it was named as *SC* [29].

Typically, the hydrothermal preparation of nano Fe_3_O_4_ particles was chosen in this preparation process [30]. 5.00 g FeCl_3_·6H_2_O and 3.68 g FeCl_2_·4H_2_O were dissolved by 50 mL distilled water in a flask, 5.0 mL polyethylene glycol aqueous solution (5%) was added into the mixed solution under vigorous stirring at 60 °C for 1 h. A certain amount of *SC* powder was added carefully into the mixed solution and the mixture was continuously stirred at 60 °C for 4 h. Then, some NaOH aqueous solution (1 mol/L) was added slowly and the solution color changed to black. The pH value of this mixed solution was detected continuously under vigorous stirring at 60 °C. The addition of NaOH aqueous solution stopped when the pH value kept stable at 11.0. After vigorous stirring at 60 °C for 3 h, the mixture aged 2 h in an ultrasound washer and dried under nitrogen protection overnight at 383 K. The black powder was washed by distilled water and anhydrous ethanol again until the washed liquid was neutral. Finally, the black powder was vacuum dried carefully at 60 °C for 12 h and it was labelled as x%-Fe_3_O_4_@SC, x represents the mass rate of Fe_3_O_4_ in the adsorbent (iron elemental content was obtained by atomic absorption spectrometry method, and the Fe_3_O_4_ content was calculated by iron elemental content).

### 2.3. Characterization

The FT-IR spectra were recorded on KBr pellets in the range of 4000–500 cm^−1^ using a PerkinElmer spectrum One (B) spectrometer. The thermal stability of the samples was analyzed by Netzsch 209C thermogravimetric (TG/DTG) with a temperature range of 303–1173 K, and a heating rate of 10 K/min under nitrogen atmosphere.

The nitrogen adsorption-desorption was carried out by the Quantachrome NOVA-2200E automated gas sorption system after the sample was pretreated at 200 °C in a vacuum for 12 h, and the specific surface areas and average pore size were calculated automatically. The X-ray diffraction (XRD) pattern of the sample was recorded by the Bruker D8 advance device with Cu-Kα radiation (λ = 1.54 A) between 20–70° (2θ) at 40 kV and 40 mA. A vibrating-sample magnetometer (VSM) (EG & G Princeton Applied Research Vibrating Sample Magnetometer, Model 155, Washington, DC, USA) was used at room temperature to characterize the magnetic properties of magnetic material microspheres. The morphology of the samples was observed by scanning electron microscope (SEM, JEOL 6500F). X-ray photoelectron spectroscopy (XPS) was used to probe the surface composition of samples using an ESCALAB250.

### 2.4. Adsorption and Desorption Study

The adsorption tests were conducted using aqueous solutions of potassium dichromate (K_2_Cr_2_O_7_) solution or petrochemical wastewater. Evaluating the pH effect and adsorption equilibrium curve were performed in the K_2_Cr_2_O_7_ solution, then many similar parameters were referred to in the petrochemical wastewater samples. The adsorbate Cr_2_O_7_^2−^ concentration was estimated by Chinese National Standards GB/T 7467-87 method [14]. (λ = 540 nm, 1,5-diphenyl carbazide is chosen as chromogenic agent).

For adsorption study, 0.03 g of the x%-Fe_3_O_4_@*SC* adsorbent was dispersed into 50 mL of chromium(VI) ions solution 80 mg/L at 298 K, pH 2, stirred for 180 min and the agitation speed was fixed at 400 rpm. The pH was adjusted using sulfuric acid or ammonia water and all pH measurements were carried out using a digital pH meter. The mixture was filtered with filter paper and residual Cr(VI) concentration in the filtrate was determined by microspectrophotometer device using a matched quartz cells. In order to assure accuracy, some samples were analyzed by atomic absorption spectrometry method (GBS, SENS AE) according to method 3110 of APHA [31].

The Fe_3_O_4_@*SC* adsorbents are used for chromium(VI) ions adsorption, and the influencing factors including the initial chromium(VI) ions concentration, adsorption time, the mass proportion of Fe_3_O_4_ and *SC*_,_ the pH value and temperature on the adsorption behavior of Fe_3_O_4_@*SC* for chromium(VI) ions were investigated. At the same time, the adsorption amount and adsorption efficiency are obtained by the following equations.

(1) $$Q_{e}=\frac{(C_{0}-C_{e})\times V}{m}$$

(2) $$R=\frac{\left(C_{0}-C_{e}\right)}{C_{0}}\times 100\%$$

where *Q_e_* (mg/g) is the adsorption amount of the adsorbent for chromium(VI) in the solution; *C*_0_ is the initial concentration of chromium(VI) in the solution before adsorption; *C_e_* is the equilibrium concentration of chromium(VI) ions (mg/L) after adsorption; *m* and *V* are the substrate mass (g) of the adsorbent and solution volume (L); *R* represents the removal rate (%) of chromium(VI) in the solution.

Additionally, for desorption study, 0.03 g of 10%-Fe_3_O_4_@*SC* was dispersed into 50 mL of chromium(VI) ions solution 80 mg/L at 298 K, pH 2, stirred for 180 min and the agitation speed was fixed at 400 rpm. Then, the saturated Cr(VI)-adsorbed adsorbent was separated from the solution. The Cr(VI) ions were desorbed from the adsorbent with 200 mL distilled water as an eluent. Then, the desorbed adsorbent was washed with 500 mL distilled water and dried, it was reused under the same conditions to study the reutilization performance of this adsorbent.

### 2.5. Data Processing

The adsorption capacity (Q_e_) was determined according to Equation (1), and the results obtained were plotted as a function of the equilibrium concentration in the liquid phase (C_e_). The equilibrium data were analyzed according to the Langmuir and Freundlich isotherm models. Two models presented in Table 1 were adjusted to the experimental data by the nonlinear regression method of the software Statistica 7.5 (Statsoft, Tulsa, OK, USA), using the Levenberg–Marquardt algorithm [32].

The adsorption tests were carried out with 50 mL of solution with pH corrected for the same conditions as the isotherms, under agitation at 400 rpm and 298 K.

## 3. Results and Discussion

### 3.1. Characterization of Fe_3_O_4_@SC

**Figure 1:**
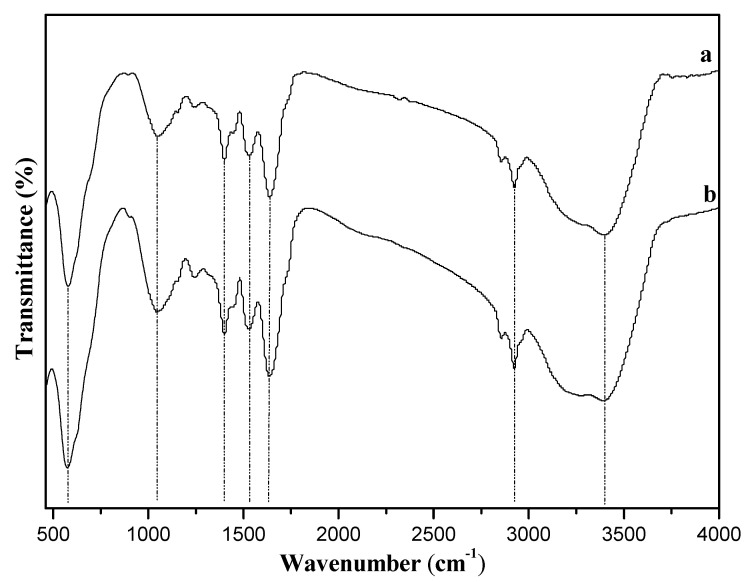
FT−IR spectra of 10%-Fe_3_O_4_@*SC* adsorbent. a fresh, b after adsorption.

FT-IR spectra of 10%-Fe_3_O_4_@*SC* adsorbent (including fresh and after adsorption) are shown in Figure 1. There are some characteristic adsorption peaks in the curves. The acute adsorption peak at 574 cm^−1^ belongs to the stretching vibration of the Fe-O bond of Fe_3_O_4_ in the adsorbent, which is in accordance with the literature result [33]. The broad adsorption peaks at 1041 cm^−1^ are ascribed to asymmetry stretching vibrations of the C-O bond in protein. The weak peaks at 1401 cm^−1^ and 1523 cm^−1^ belong to the amide II band, so the *Saccharomyces cerevisiae* became the main body of the absorbent. The acute characteristic peaks at 1635 cm^−1^ are ascribed to a combination of simultaneous C=O and N-H vibrations (amide I band). There are some small peaks near 2921 cm^−1^ belonging to the stretching vibrations of C-H in -CH_3_, -CH_2_ or -CH. The large and broad peaks near 3375 cm^−1^ belong to stretching vibrations of -NH_2_ and -OH in protein structure, which is in accordance with the literature result [34].

After adsorption of Cr(VI), the peak near 1041 cm^−1^ broadens smoothly and may be assigned to the interaction of oxygen from *SC* with Cr(VI) ions. The peak near 1635 cm^−1^ is stronger, maybe due to the weak complexation between the carboxylic group and Cr(VI) ions. The peaks near 3375 cm^−1^ broaden distinctly, partially due to complexation of hydroxy groups with Cr(VI). Especially, there are dilated peaks near 3100 cm^−1^ that are ascribed to the stretching vibrations of the Cr-O bond, but the signal is interfered by the intense stretching vibration signals of -NH_2_ and -OH in the protein structure, which are in accordance with the literature result [35]. So, the *Saccharomyces cerevisiae* participated in the adsorption process for the Cr(VI) ions.

**Figure 2:**
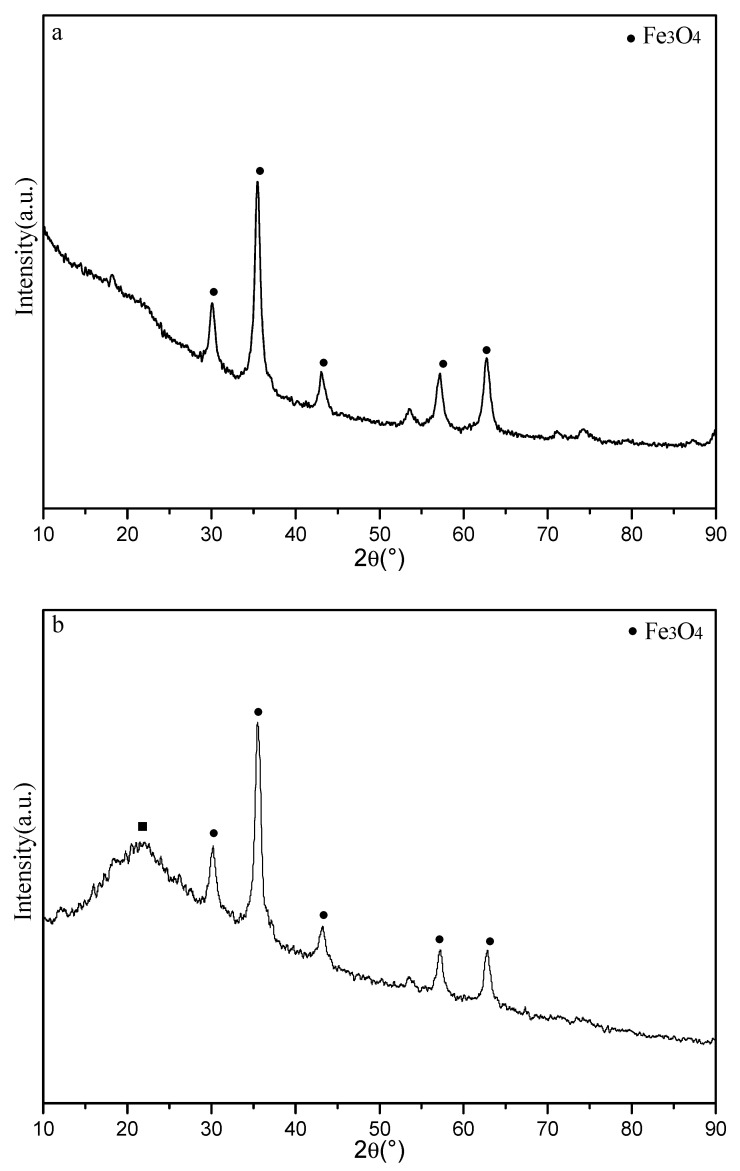
X-ray diffraction (XRD) patterns of 10%-Fe_3_O_4_@*SC* adsorbent. (**a**) fresh, (**b**) after adsorption.

The XRD spectra of the samples (including fresh and after adsorption) are shown in Figure 2. The characteristic diffraction peaks at 2θ = 30.2°, 35.5°, 43.2°, 53.5°, 57.2° and 62.7° are corresponding to (220), (311), (400), (422), (511) and (440) crystalline planes of Fe_3_O_4_ species [36]. The complex composition of these crystal planes shows Fe_3_O_4_ cubic crystal characteristics in the adsorbent [37]. The average particle size of Fe_3_O_4_ species was nearly 44.8 nm, which was calculated from the XRD data based on Scherrer equation. We can’t find obvious changes about the characteristic diffraction peaks of Fe_3_O_4_ species after adsorption, but there is a new broad peak at 2θ = 22.3°, which may be ascribed to the strong adsorption behavior between Fe_3_O_4_@*SC* adsorbent and Cr(VI) ions in water solution. Hence, the crystal structure retains original features after adsorption.

The TG and DTG analysis of some samples are shown in Supplementary Figure S1. The thermal stability of the 10%-Fe_3_O_4_@*SC* adsorbent is not good, so this nanomaterial can only be used at room temperature. There are not obvious differences for this adsorbent after adsorption. The vibrating sample magnetometer (VSM) is used for testing the magnetic properties of Fe_3_O_4_ and 10%-Fe_3_O_4_@*SC* at room temperature, respectively. It is clear that the magnetization saturation (Ms) of 10%-Fe_3_O_4_@*SC* is lower than standard Fe_3_O_4_ particles from Figure S2. Although the Ms value of 10%-Fe_3_O_4_@*SC* is low, the elegant magnetic feature is still certified.

**Figure 3:**
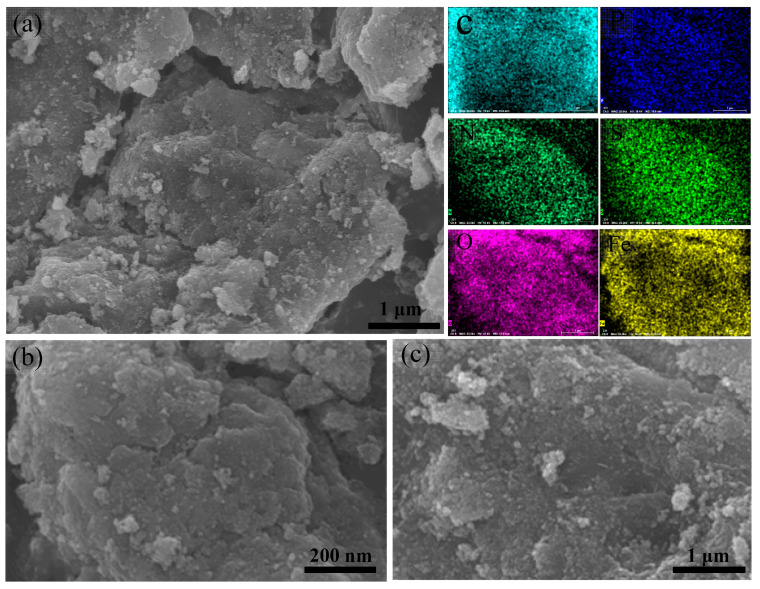
SEM and element distribution images of 10%-Fe_3_O_4_@SC adsorbent. (**a**,**b**) The micro morphology of fresh adsorbent at different magnifications. (**c**) The micro morphology of adsorbent after adsorption.

The SEM images of 10%-Fe_3_O_4_@*SC* and *SC* adsorbents are displayed in Figure 3 and Figure S3, respectively. The irregular layered structure characteristics of fresh absorbent is shown by Figure 3a. We can see the Fe_3_O_4_ particles have been coated in *SC* basal body from Figure 3b. The color element distribution diagram shows the C, N, O, P, S and Fe elements are dispersed very homogeneously on the surface of fresh absorbent. Figure 3c shows small particles were congregated weakly on the surface of the adsorbent after adsorption, which is due to the strong adsorbance behavior between active sites and Cr(VI) ions in water solution. We also see micro particle shapes of the *SC* material in Figure S3. Numberless coral pellets are accumulated on the surface of the *SC* material, which shows the disorder feature. Hence, the magnetism is beneficial to the orderly optimization of the micro surface.

The textural properties of BET surface area, average pore size, pore volume and average particle size of the samples are shown in Table S1. Both 3%-Fe_3_O_4_@*SC* and 7%-Fe_3_O_4_@*SC* adsorbents have a relatively large average pore size of about 17.6 nm and larger surface area of about 179–181 m^2^/g. With the increment of Fe_3_O_4_ mass ratio in the adsorbent, both average pore size and pore volume obviously cut down, which was ascribed to too many Fe_3_O_4_ particles blocking some pore channels. The 10%-Fe_3_O_4_@*SC* absorbent presents the medium average pore size, pore volume and average particle size, and the largest BET surface area is 182.54 m^2^/g in the x%-Fe_3_O_4_@*SC* absorbents.

### 3.2. Effect of pH Value in Water Solution

**Figure 4:**
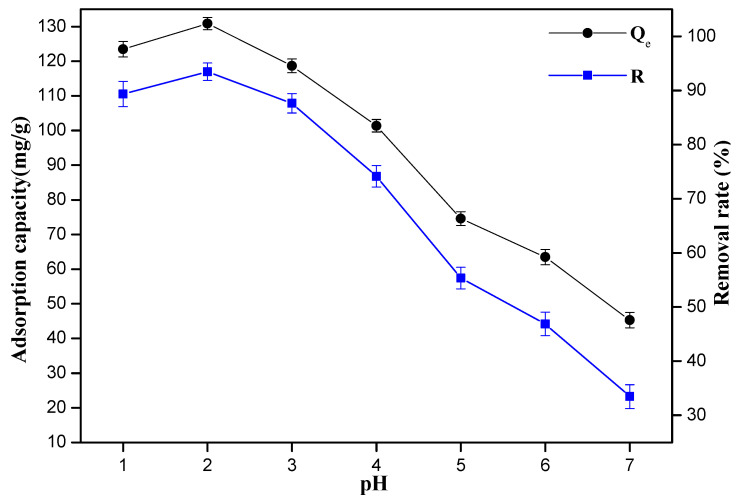
Effect of pH value on the sorption of Cr(VI) ions.

The pH value in the water solution is a very important parameter for the adsorption of Cr(VI) ions, and the variation trend of the removal rate and the adsorption capacity of the 10%-Fe_3_O_4_@*SC* adsorbent are shown in Figure 4. Both the adsorption capacity of adsorbent and the removal rate of Cr(VI) ions decreased with the increment of pH value. Amine groups on the surface of 10%-Fe_3_O_4_@*SC* absorbent were protonated to be -NH_3_^+^ easily in a high concentration of H^+^ solution, so the chelating reaction might be more difficult. The chromate(VI) anions might be electrostatically attracted to -NH_3_^+^ centers, then the reduction or chemical adsorption occurred easily. The adsorption capacity of adsorbent and the removal rate of Cr(VI) ions in water solution with pH of 1.0 are not better than the corresponding situation with a pH of 2.0. Considering the hydrolysis reaction of Cr(VI) ions may occur easily in alkaline or weak acidic solution, the optimal pH value of water solution was 2.0 and this parameter was fixed for further investigation.

Cr(VI) ions are an oxidizer and their oxidizing properties are stronger in acidic solutions, and the reduction of Cr(VI) to Cr(III) maybe interferes with the adsorption behavior. Some adsorption experiments were finished at low pH and the results are listed in Table S2. We found that the absolute error of data obtained from the diphenylcarbazide method and atomic absorption spectrometry was small at a lower pH. Especially, the results are the same at pH = 7 exactly, so the reduction of Cr(VI) to Cr(III) only occurs in acidic solutions. We also found that the reduction of Cr(VI) might occurred after adsorption from Table S2. Compared to the adsorption capacity, the reducing performance of the adsorbent is relative weak, but the adsorption and reduction process occurred in the petrochemical wastewater containing Cr(VI) ions.

### 3.3. Effect of Initial Cr(VI) Ions Concentration

**Figure 5:**
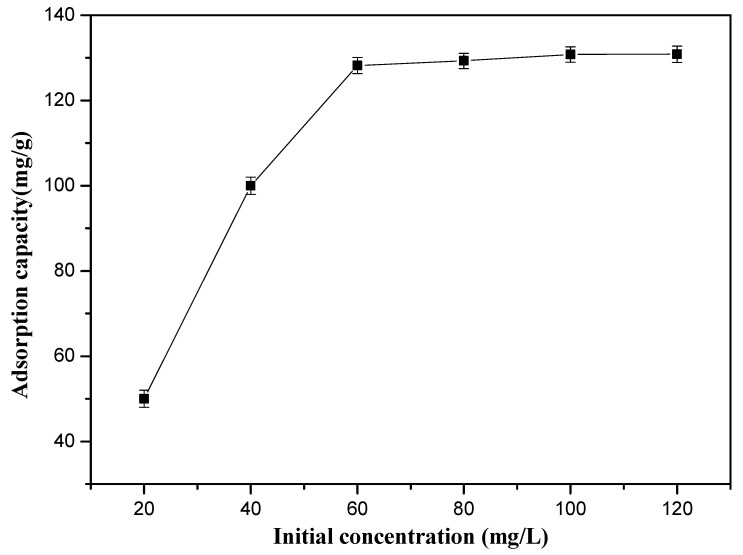
Effect of initial Cr(VI) ions concentration in solution on the sorption.

The initial Cr(VI) ions concentration is a very important parameter for the adsorption, and the adsorption capacity of the 10%-Fe_3_O_4_@*SC* adsorbent is described with the range from 20 to 120 mg/L in Figure 5. We found a clear upward trend with the increment of initial Cr(VI) ions concentration. The adsorption capacity of the 10%-Fe_3_O_4_@*SC* adsorbent was almost unchanged since the initial Cr(VI) ions concentration was more than 80 mg/L.

In a low concentration of Cr(VI) ions solution, there were many high active sites on the surface of the 10%-Fe_3_O_4_@*SC* adsorbent, which were more than the amount of initial Cr(VI) ions in the solution, so the collision between Cr(VI) ions and active sites increased, and the adsorption capacity of the 10%-Fe_3_O_4_@*SC* adsorbent also increased distinctly. In a high concentration of Cr(VI) ions solution, the amount of active sites on the surface of the 10%-Fe_3_O_4_@*SC* adsorbent were not adequate, and it was difficult for the adsorption to so many Cr(VI) ions in the solution, so the curve tended to stabilize.

### 3.4. Effect of Fe_3_O_4_ Loading

The different adsorption capacities of adsorbent and the removal rate of Cr(VI) ions in solution are listed in Table 2. It was clear that the adsorption capacity of adsorbent was different with the content of *SC* and the loading of Fe_3_O_4_ (wt.%). The maximum adsorption capacity of adsorbent reached 128.03 mg/g due to the abundant groups such as amino, hydroxyl and ketone on the surface of adsorbent, and the removal rate of Cr(VI) ions in solution reached 96.02%, so the major Cr(VI) ions could be removed effectively. Too much Fe_3_O_4_ loading was not suitable due to some magnetic particles blocking the pores in the microstructure. The mass of organic matter decreased with the increment of Fe_3_O_4_ loading, so the adsorption capacity of organic matter (Table 2) was calculated excluding the mass of Fe_3_O_4_ in the adsorbent. We found the gap of adsorption performance became smaller. Hence, the appropriate magnetism improved the adsorption capacity of organic matter.

The adsorption performance of different adsorbents consists with the textural properties of adsorbents (Table S1), and larger BET surface area and larger pore volume can improve the adsorption capacity of the adsorbent. The strength of magnetism is determined by Fe_3_O_4_ loading, and we also found too weak magnetism was not suitable, so the best mass proportion of *SC* and Fe_3_O_4_ in the adsorbent is 90%:10%. The data were obtained from atomic absorption spectrometry were lower than the results, which indicated a small amount of Cr(VI) ions might be reduced into Cr(III) ions, so both adsorption and reducing capacity could be reflected in this process.

### 3.5. Adsorption Isotherm Analysis

In order to explore the adsorption essence and characteristic of the 10%-Fe_3_O_4_@SC adsorbent in detail, Langmuir and Freundlich isotherm models are used to investigate this adsorption behavior when the adsorption attains dynamic equilibrium. Test data fitting the curve of Langmuir and Freundlich adsorption isotherm model are shown in Figure S4 and Figure S5, respectively. We found the experimental data were fitted well by the Langmuir and Freundlich adsorption isotherm models, but the linear correlation performances were different. The linear form equations of the Langmuir and Freundlich models are expressed as follows [38].

$$\frac{C_{e}}{Q_{e}}=\frac{1}{Q_{m}K_{L}}+\frac{C_{e}}{Q_{m}}\;\ln Q_{e}=\ln K_{F}+\frac{1}{n}\ln C_{e}$$

where *C_e_* (mg/L) is the Cr(VI) ions concentration, *Q_e_* (mg/g) is the uptake amount of Cr(VI) ions at equilibrium and *Q_m_* (mg/g) represents the mono-layer maximum absorption of the absorbents. K_L_ and K_F_ represent Langmuir constant and Freundlich constant respectively, and n is the adsorption index.

Additionally, the separation factor (*R_L_*) is calculated by the following equation [39], which can distinguish whether the adsorption is favorable or not.

$$R_{L}=\frac{1}{1+C_{0}K_{L}}$$

where, the adsorption process is favorable since the *R_L_* value is in the range of 0 to 1, the adsorption process is lineal or unfavorable since the *R_L_* = 1 or *R_L_* > 1.

**Figure 6:**
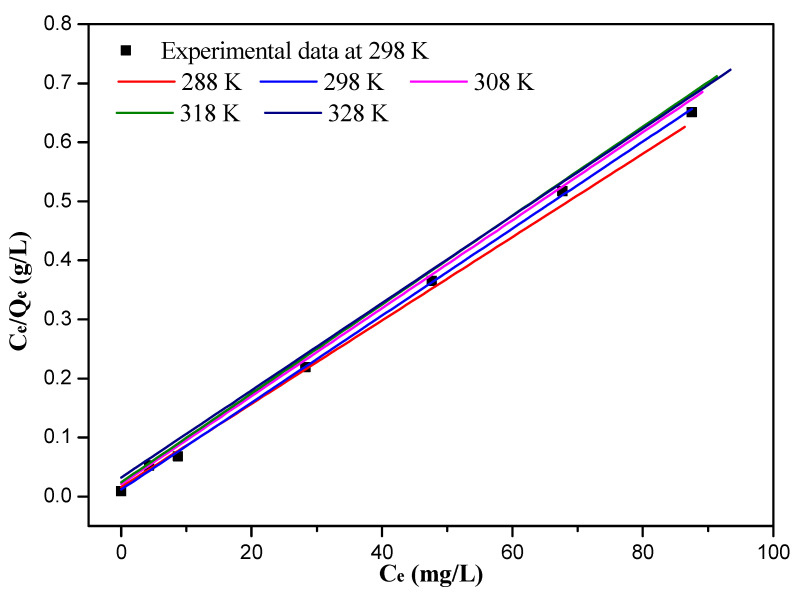
Experimental data at 298 K and Langmuir isotherm plots for the adsorption of Cr(VI) ions at different temperatures.

**Figure 7:**
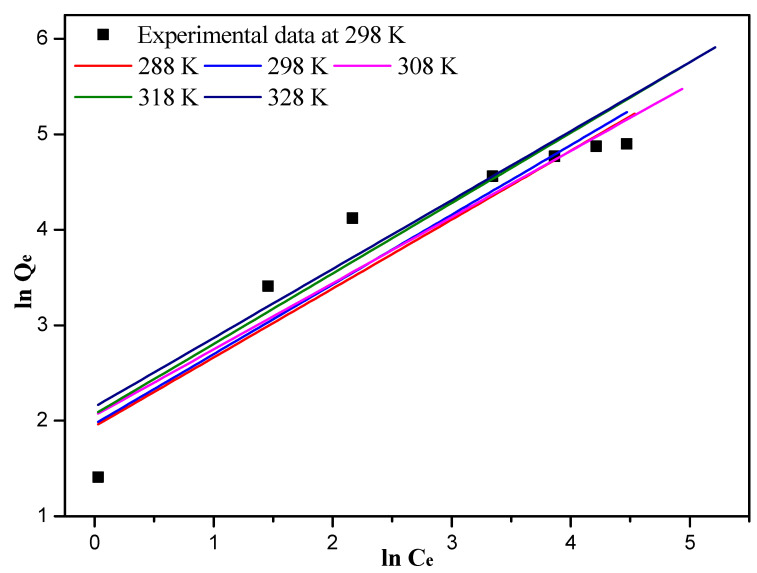
Experimental data at 298 K and Freundlich isotherm plots for the adsorption of Cr(VI) ions at different temperatures.

The experimental data of Langmuir and Freundlich isotherm fitting at different temperatures were listed in Table S3 and Table S4, respectively. The experimental data at 298 K and Langmuir isotherm fitting diagram at different temperatures are shown in Figure 6. The experimental data at 298 K and Freundlich isotherm fitting diagram at different temperatures are shown in Figure 7. Compared to the Freundlich isotherm model, the R^2^ value of Langmuir model is better (0.9993 > 0.8779).

Langmuir and Freundlich model fitting parameters are listed in Table 3, and the correlation coefficients of the Freundlich model with the different temperatures are poor (0.8697~0.8779). The maximum adsorption capacity of the 10%-Fe_3_O_4_@*SC* adsorbent is 128.03 mg/g, and the theoretical maximum adsorption capacity of the 10%-Fe_3_O_4_@*SC* adsorbent is 135.68 mg/g at the temperature of 298 K. The error is small, and the relative error is approx. 5.96%, so this gap can be ignored, and the Langmuir isotherm model could describe the isotherm adsorption behavior better. 

The values of the R_L_ were 0.0270, 0.0194, 0.0338, 0.0382 and 0.0515 at the temperature of 288 K, 298 K, 308 K, 318 K and 328 K in the Langmuir model analysis, respectively. All of the values of R_L_ are less than 1.0, indicating that 10%-Fe_3_O_4_@*SC* was an effective adsorbent for Cr(VI) ions adsorption in solution. This adsorption belongs to a monolayer adsorption of active centers (hydroxyl, amino), which was not interfered by the adsorbed quantity and energy.

Recently, many new materials such as MOFs were prepared for the adsorption of Cr(VI) ions in water solution, and the maximum adsorption capacity for Cr(VI) ions in water solution by the typical adsorbents were listed in Table 4. The advantage of this adsorbent was distinct and affirmative due to the adsorption capacity for Cr(VI) ions reached 128.03 mg/g, and all these experiments were performed by diluted sample from petrochemical wastewater. Hence, this adsorbent could be applied for the removal of Cr(VI) ions in petrochemical wastewater.

### 3.6. Effect of Adsorption Time and Adsorption Kinetics

**Figure 8:**
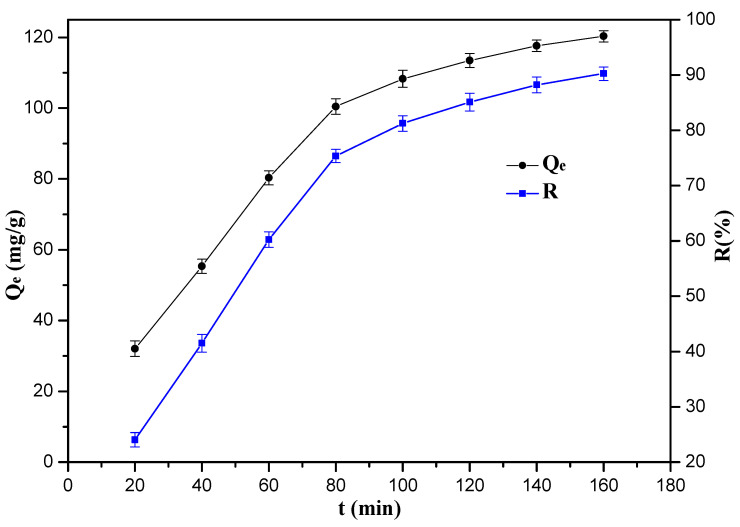
Effect of adsorption time on the uptake of Cr(VI) ions.

Effect of adsorption time on the uptake of Cr(VI) ions by the 10%-Fe_3_O_4_@*SC* adsorbent is shown in Figure 8. When the adsorption time is below 80 min, the adsorption capacity of the 10%-Fe_3_O_4_@*SC* adsorbent increases distinctly. However, when the adsorption time exceeds 80 min, the removal rate of Cr(VI) ions increases slowly and maintains the maximum value. The amount of many active adsorb sites (such as –NH_2_, –OH) are limited, and Cr(VI) ions could be adsorbed by these sites rapidly at 0–80 min, but the reduction of active adsorb sites can decrease adsorption rate at 80–160 min directly. The adsorption capacity value was almost unchanged when the adsorption time exceed 160 min, so the adsorption had reached the dynamic equilibrium.

The adsorption capacity (Q_t_) was fitted with pseudo-first-order or pseudo-second-order method with the same time intervals, and the calculated models are expressed in Equations [45].

$$\ln(Q_{e}-Q_{t})=\ln Q_{e}-k_{1}t\;\frac{t}{Q_{t}}=\frac{1}{k_{2}Q_{e(cal)}^{2}}+\frac{1}{Q_{e(cal)}}t$$

where, *Q_t_* and *Q_e_* (mg/g) are the adsorption capacity at time t and equilibrium time, respectively. *t* (min) is adsorption time, *k*_1_ is the pseudo-first-order model rate constant, and *k*_2_ is the pseudo-second-order model rate constant.

**Figure 9:**
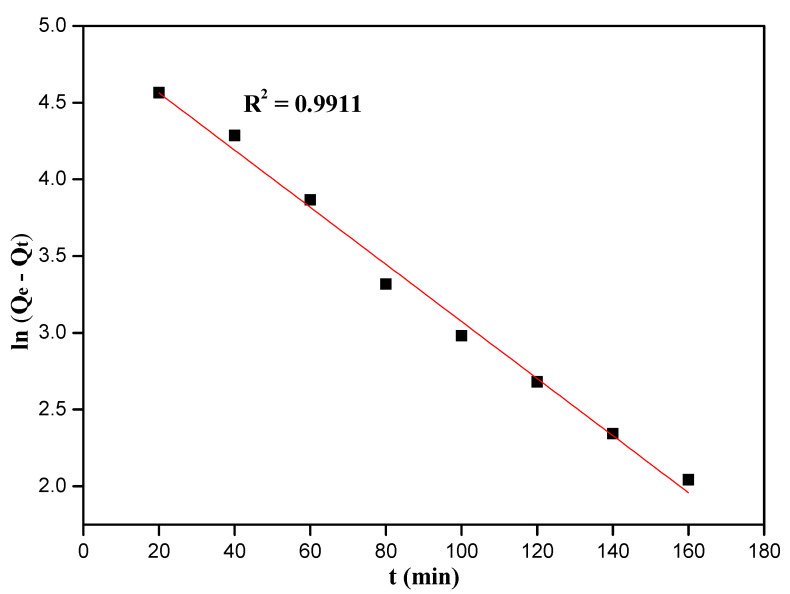
Fit of kinetic data to pseudo-first-order model.

**Figure 10:**
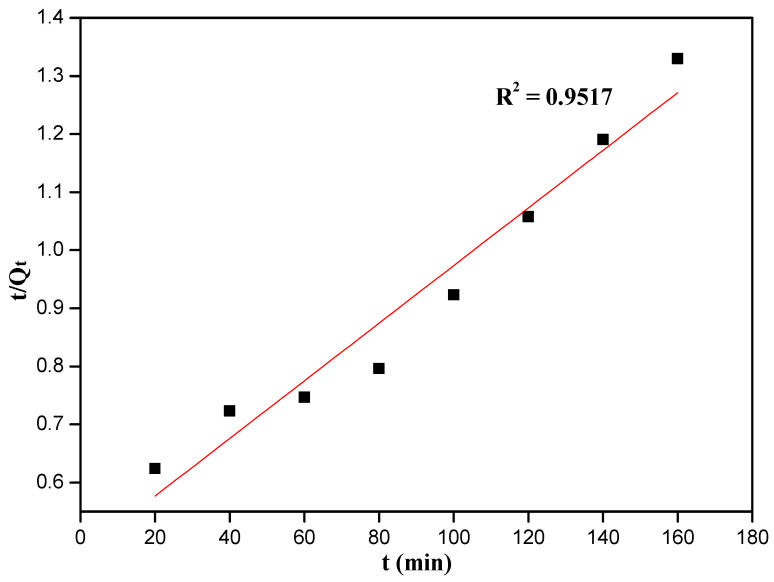
Fit of kinetic data to pseudo-second-order model.

Fitting of kinetic data to pseudo-first-order and pseudo-second-order models is displayed in Figure 9 and Figure 10, respectively. We found the relative linear coefficient of pseudo-first-order was better than pseudo-second-order model (0.9911 > 0.9517), and the pseudo-first-order kinetic model was preferred to describe this adsorption kinetic system. Many kinetic parameters such as R^2^, k_1_, k_2_ and Q_e(cal)_ were listed in Table S5. The calculated adsorption capacity Q_e(cal)_ of the 10%-Fe_3_O_4_@*SC* adsorbent from the pseudo-first-order kinetic model analysis was closer to the really experimental value, so the physical adsorption and chemisorption might coexist, and the chemisorption was inapparent. Cr(VI) ions from water solution to the surface of adsorbent were controlled by the diffusion step, so the stirring or oscillation was beneficial to this adsorption.

### 3.7. Effect of Temperature and Thermodynamic Analysis

The adsorption experiments for Cr(VI) ions using the 10%-Fe_3_O_4_@*SC* adsorbent were investigated at many different temperatures (from 288 to 328 K) and experiments are listed in Table 5. The high temperature might not be helpful for the adsorption of Cr(VI) ions, and the adsorption rate was low at low temperature, so 298 K was the optimal temperature. Consider the hydrolysis of Cr(VI) ions occurred easily at high temperature, so the low temperature was suitable to this adsorption by this adsorbent. Additionally, the thermodynamic parameters of the 10%-Fe_3_O_4_@*SC* adsorbent were obtained by the Van’t Hoff equation, and ΔH^0^ represented the enthalpy change, ΔS^0^ represented the entropy change and ΔG represented the Gibbs free energy change.

$$\ln(\frac{q_{e}}{C_{e}})=-\frac{\Delta H^{0}}{RT}+\frac{\Delta S^{0}}{R}\;\Delta G=-RT\cdot \ln(\frac{Q_{e}}{C_{e}})$$

The plot dependence of ln(*Q_e_*/*C_e_*) on 1/*T* is displayed in Figure S6, and the correlation coefficient is R^2^ = 0.9291 by the data analysis. The value of Δ*H*^0^ and Δ*S*^0^ in the above equation were −62.489 kJ/mol and −180.730 J/mol·K by calculation, and all Δ*G* (Table 5) and *ΔH*^0^ values were negative at different temperatures, so the adsorption Cr(VI) ions process of this adsorbent was spontaneous and exothermic.

### 3.8. Mechanism for Adsorption of Cr(VI) Ions

The Cr(VI) adsorption by this adsorbent may include physical adsorption and chemisorption. There are many actions such as chemical bonding, coordination bonding, acid-base interactions or ion exchange for the chemisorption. There are three kinds of existing forms for Cr(VI) ions in the solution when the solution is acidic, and the following transitions are inevitably [46].

Cr_2_O_7_^2−^ + H_2_O ⇌ 2 HCrO_4_^−^ K_a_=10^−2.2^

HCrO_4_^−^ ⇌ CrO_4_^2−^ + H^+^ K_a_=10^−6.49^

The adsorption mechanism of Cr(VI) ions can be described in four steps: (1) protonation of active groups on the surface of the adsorbent; (2) adsorption of the metal ions on the protonated substrate and the metal ionic complexation; (3) Cr(VI) ions can be reduced by means of electron donor groups; and (4) chemical complexation, electrostatic attraction or cation exchange process are finally taking place [47]. The organic component of *SC* was the primary adsorbent of Cr(VI) ions, which is certified by adsorbent characterization (FT-IR, XRD and SEM).

Coordination bonds might be formed between nonmetallic elements and Cr(VI) ions due to the *SC* material having abundant nonmetallic elements such as N, O and S. However, the coordination capacity of Cr(VI) ions was limited and the steric effect of oxygen atoms in Cr_2_O_7_^2−^, HCrO_4_^−^ or CrO_4_^2−^ ions. Another likely adsorption mechanism was ion exchange because the solution was acidic and there are many groups such as -NH_3_^+^ on the surface of the adsorbent.

**Figure 11:**
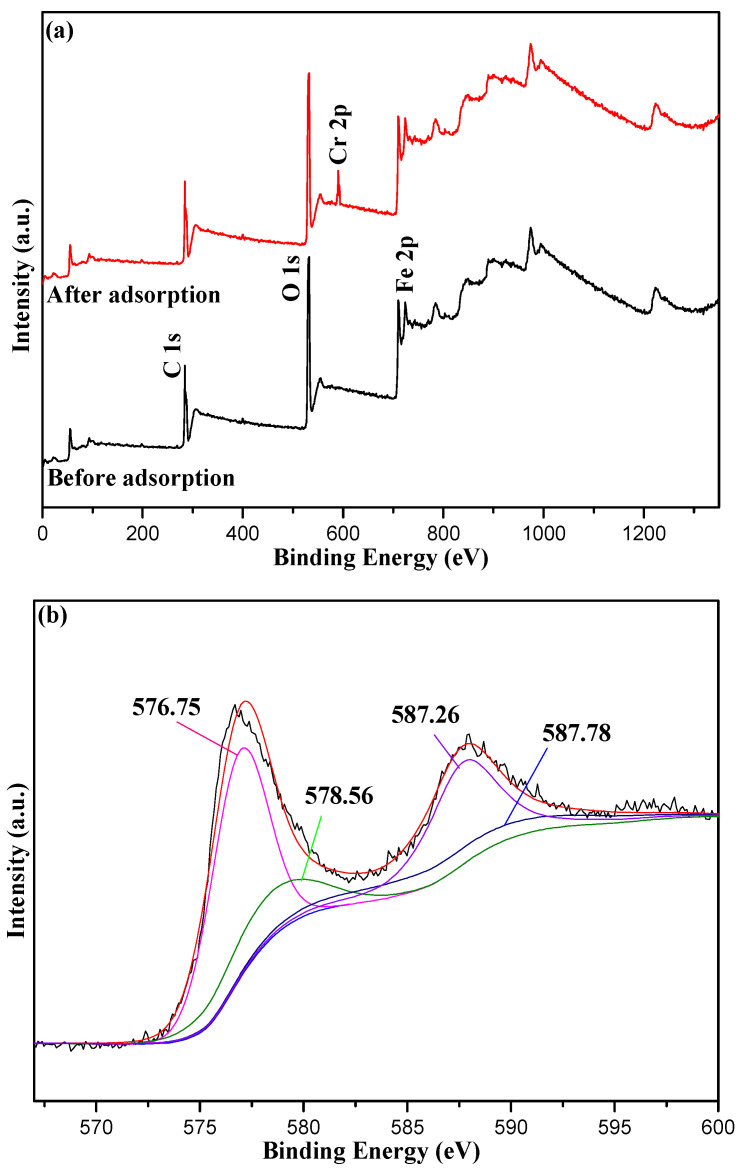
XPS spectra of the 10%-Fe_3_O_4_@*SC* adsorbent. (**a**) Full-scan; (**b**) High-resolution of Cr 2p after adsorption.

XPS spectra of adsorbent samples are shown in Figure 11. It was clear that Cr(VI) ions had been adsorbed successfully from the Full-scan XPS spectra in Figure 11a because the new peak that appeared at 587.0 eV belonged to the Cr-2p energy band. Additionally, the peaks at 576.75 and 587.26 eV were corresponding to the Cr 2p_3/2_ orbital peak, which were considered to be Cr(III) [48], and the peaks at 578.56 and 587.78 eV, corresponding to the Cr 2p_1/2_ orbital peak, were considered to be Cr(VI) (Figure 11b). A portion of Cr(VI) was rapidly reduced to Cr(III) after being adsorbed by this adsorbent under acidic conditions, so the ion exchange occurred successfully in the adsorption.

The chemical bonds between Cr(VI) ions and active groups might be weak, which was consistent with there not being any significant corresponding bond vibration signal in FT-IR spectra (Figure 1). The acid-base interactions might occur, but the process was so complex that there were no evident evaluation [46]. The value of pH was almost not changed after adsorption as the mass of adsorbent was only 0.03 g, so this interaction can only be confirmed depending on new technologies in the future. Hence, this adsorption process was complicated, and all the performances conformed to the characteristic of the biosorbent.

### 3.9. Effect of Interfering Ions

**Figure 12:**
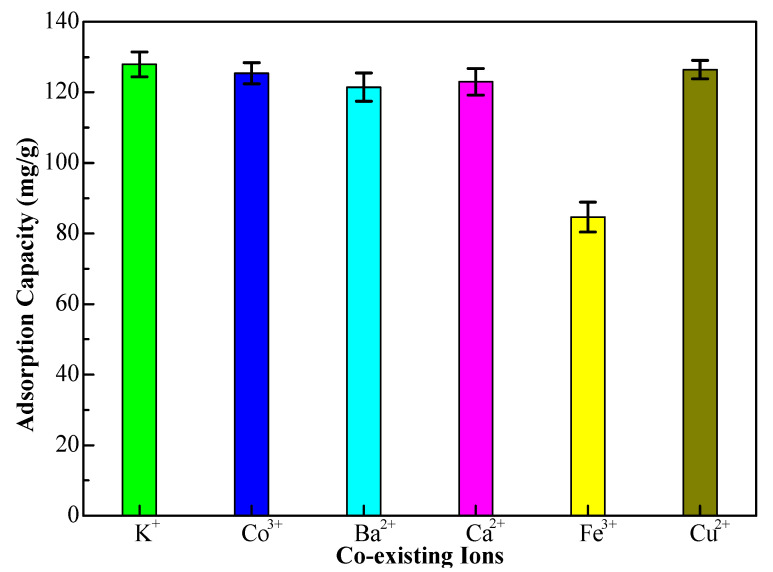
The adsorption capacity of 10%-Fe_3_O_4_@*SC* adsorbent for Cr(VI) ions under the coexistence ions.

In petrochemical wastewater, there are many metal ions that may compete with Cr(VI) ions in the adsorption process, so the competitive adsorption experiments of interfering ions need to be investigated. Many coexisting cations such as K(I), Co(III), Ba(II), Ca(II), Fe(III), and Cu(II) were mixed with the Cr(VI) ions solution, respectively (interfering ions concentration, 80 mg/L). The different adsorption capacities of the 10%-Fe_3_O_4_@*SC* adsorbent for Cr(VI) ions are displayed in Figure 12. The interference of Fe(III) ions was serious, maybe due to the magnetized property of the iron element, and the adsorption capacity of the 10%-Fe_3_O_4_@*SC* adsorbent for Cr(VI) ions was as low as 84.67 mg/g. We can observe the adsorption capacity of Cr(VI) ions is almost not changed distinctly to the other coexisting ions, so the competitive effect is selectivity. Thereby the selectivity adsorption to Cr(VI) ions has been affirmed and iron ions should be removed before the adsorption.

### 3.10. Reutilization of the 10%-Fe_3_O_4_@SC Adsorbent

**Figure 13:**
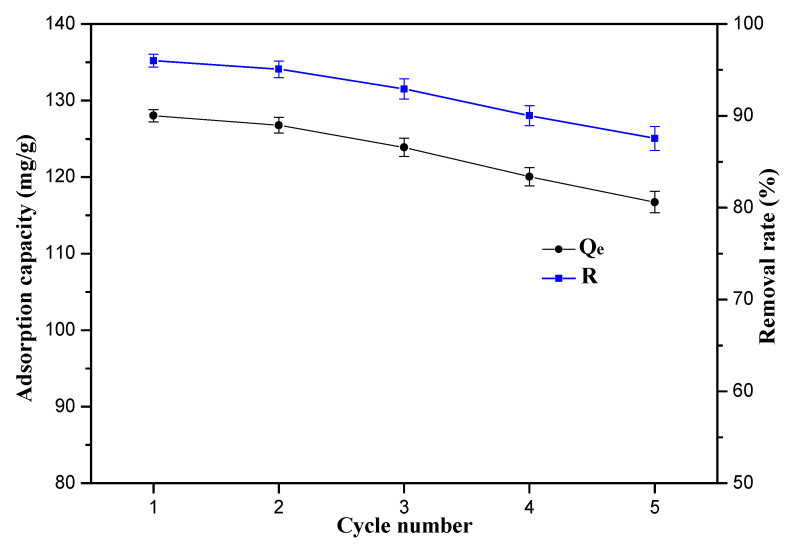
Repeated use of the 10%-Fe_3_O_4_@*SC* adsorbent.

In order to evaluate economic value and application prospects, the recycling performance of the 10%-Fe_3_O_4_@*SC* adsorbent are displayed in Figure 13. After the adsorption in Cr(VI) ions solution, adsorbent could be transferred into eluent solution. The removal rate of Cr(VI) ions by this adsorbent was still as high as 87.54%, and the adsorption capacity of Cr(VI) ions by this adsorbent was sill as high as 116.72 mg/g after four cycles. The recycling adsorption performance by the *SC* adsorbent is listed in Table S6. We can find that both the adsorption capacity of adsorbent and removal rate of Cr(VI) ions cut down quickly. Hence, the magnetic property is beneficial for the development of cyclic use capacity.

Actually, the physical adsorption, chemisorption and reduction occurred in the solution, and it was difficult for us to control. For the physical adsorption, the desorption was easy to occur in the eluent. For the chemisorption process, the desorption was difficult to occur, but the reduction between Cr(VI) ions and *SC* was beneficial to the transfer of Cr(VI) to Cr(III). The reduced form as Cr(III) was bonded to the adsorbent weakly in acid conditions and released into the solution easily. So the good desorption efficiency can be shown and the stronger reducing power can support the good cycling adsorption. Hence, the good desorption capacity and good reuse capacity of this magnetic adsorbent had been affirmed, and it showed great application prospect in the removal of Cr(VI) ions in petrochemical wastewater.

## 4. Conclusions

As the efficient adsorbent for the adsorption of Cr(VI) ions in petrochemical wastewater, the Fe_3_O_4_@*SC* adsorbents were prepared successfully. The physicochemical properties of adsorbents were characterized by FT-IR, XRD, TG, VSM, SEM, BET and XPS technologies. The results showed that homogeneous dispersal, large surface area and good thermal stability. *Saccharomyces cerevisiae* participated in the adsorption process for the Cr(VI) ions, and the adsorption performance was better since the magnetic particles were involved in the adsorbent. The maximum adsorption capacity of the 10%-Fe_3_O_4_@*SC* adsorbent was 128.03 mg/g and the corresponding removal rate of Cr(VI) ions was 96.02% under the optimized conditions. The Langmuir model was better for the adsorption isotherm analysis than the Freundlich model, and the correlation coefficient was as high as 0.9987, so the feature of monolayer adsorption was affirmed. This adsorption process of Cr(VI) ions by the 10%-Fe_3_O_4_@SC adsorbent was spontaneous and exothermic by thermodynamic data analysis. The reutilization performance of this new adsorbent was good, and the adsorption capacity of this adsorbent still was more than 110.0 mg/g after five recycle experiments, so the good desorption and reuse performance were affirmed, and the application prospects are huge. The pseudo-first-order kinetic model was preferred to describe this adsorption kinetic system better, and the physical adsorption and chemisorption might coexist. The chemisorption was inapparent and Cr(VI) ions from solution to the surface of adsorbent were controlled by the diffusion step, so the stirring or oscillation was beneficial to this adsorption. The advantage of this adsorbent was distinct and affirmative as the adsorption capacity for Cr(VI) ions was better than many MOFs adsorbents. In the mechanism analysis, rapid adsorption preferential occurred by the organic component of SC, and a portion of Cr(VI) ions might be rapidly reduced to Cr(III) after being adsorbed by this adsorbent in acidic solution, so the ion exchange occurred successfully. All in all, the adsorption process was complicated and all the performances conformed to the characteristics of the biosorbent. These research results indicated that the 10%-Fe_3_O_4_@*SC* adsorbent was highly efficient for Cr(VI)-ions cleanup in petrochemical wastewater solutions.

## Figures and Tables

**Table 1:** Equations of the isotherm models used in this study.

Model	Equation	Parameters
Langmuir	$Q_{e}=\frac{Q_{m}K_{L}C_{eq}}{1+K_{L}C_{eq}}$	Q_m_ (mg/g) maximum adsorption capacity; K_L_ (L/mg) Langmuir constant.
Freundlich	$Q_{e}=K_{F}C{}_{e}^{1/n}$	1/n (-) heterogeneity factor; K_F_ Freundlich constant.

**Table 2:** The adsorption performance by different adsorbents.

Absorbents	Adsorption Capacity (mg/g)	Removal Rate of Cr(VI) Ions (%)	Adsorption Capacity ^a^ (mg/g)	Adsorption Capacity of Organic Matter ^b^ (mg/g)
*SC*	100.42	75.32	97.49	100.42
3%-Fe_3_O_4_@SC	120.15	90.11	116.31	123.86
7%-Fe_3_O_4_@SC	124.61	93.46	120.24	133.99
10%-Fe_3_O_4_@SC	128.03	96.02	122.47	142.26
15%-Fe_3_O_4_@SC	110.32	82.74	104.82	129.79
20%-Fe_3_O_4_@SC	87.46	65.60	79.34	109.33

Adsorption conditions: the initial Cr(VI) ions concentration, 80 mg/L; volume, 50 mL; pH, 2.0; adsorbent mass, 0.03 g; temperature, 25 °C; adsorption time, 3 h; agitation speed, 400 rpm. ^a^ The data were analyzed from atomic absorption spectrometry method. ^b^ The data were calculated by the mass of organic matter (excluding the mass of Fe_3_O_4_).

**Table 3:** Langmuir and Freundlich model fitting parameters.

Temperature /K	Langmuir Model			Freundlich Model		
	Q_m_/(mg/g)	K_L_/(L/mg)	R^2^	K_F_/(mg/g) (L/mg)^1/n^	n	R^2^
288	141.64	0.450	0.9975	6.97	1.38	0.8719
298	135.68	0.633	0.9993	7.16	1.37	0.8779
308	134.23	0.358	0.9951	7.80	1.44	0.8519
318	132.80	0.314	0.9915	7.91	1.36	0.8705
328	135.32	0.230	0.9927	8.55	1.38	0.8697

Adsorption conditions: the initial Cr(VI) ions concentration, 80 mg/L; volume, 50 mL; pH, 2.0; adsorbent mass, 0.03 g; adsorption time, 3 h; agitation speed, 400 rpm.

**Table 4:** Comparison of adsorption capacity for Cr(VI) ions in water solution.

Adsorbents	Q _max_ (mg/g)	Refs.
ZIF-67	15.3	[40]
Fe_3_O_4_@MIL-100(Fe)	18.0	[41]
Chitosan-MOF	94.2	[42]
TMU-30	145.1	[43]
ZJU-101	245.0	[44]

**Table 5:** Effect of temperature and the Gibbs free energy change at different temperatures.

Temperature (K)	Adsorption Capacity (mg/g)	Removal Rate of Cr(VI) Ions (%)	ΔG (kJ/mol)
288	120.72	90.54	−6.63
298	128.03	96.02	−9.15
308	114.79	86.09	−5.98
318	106.84	80.13	−5.04
328	91.37	68.53	−3.51

Adsorption conditions: the initial Cr(VI) ions concentration, 80 mg/L; volume, 50 mL; pH, 2.0; adsorbent mass, 0.03 g; adsorption time, 3 h; agitation speed, 400 rpm.

## Data Availability

Data is contained within the article or Supplementary Materials. The data presented in this study are available in Supplementary Materials.

## Supplementary Materials

The following supplementary materials can be downloaded at: https://www.mdpi.com/article/10.3390/nano12183250/s1, Figure S1: The TG and DTG analysize of 10%-Fe_3_O_4_@*SC* adsorbent. Figure S2: The VSM analysis of Fe_3_O_4_ and 10%-Fe_3_O_4_@*SC* sample. Figure S3: SEM image of *SC* adsorbent; Table S1: Textural properties of SC and x%-Fe_3_O_4_@*SC* absorbents; Table S2: The adsorption performance by 10%-Fe_3_O_4_@*SC* adsorbent at lower pH; Figure S4: Test data fitting curve of Langmuir adsorption isotherm model; Figure S5: Test data fitting curve of Freundlich adsorption isotherm model; Table S3: The experimental data of Langmuir isotherm at different temperatures; Table S4: The experimental data of Freundlich isotherm at different temperatures; Table S5: Parameters of adsorption kinetic and intraparticle diffusion model; Figure S6: The plot dependence of ln(Q_e_/C_e_) on 1/T.
