# Supplementary material for: Efficient Removal of Cr(VI) Ions in Petrochemical Wastewater Using Fe3O4@Saccharomyces cerevisiae Magnetic Nanocomposite
Source: Nanomaterials (Basel). 2022 Sep 19;12(18):3250. doi: 10.3390/nano12183250 (PMC9500928; doi:10.3390/nano12183250)
Supplement: Supplementary file 1 [file nanomaterials-12-03250-s001.zip › nanomaterials-1872434-supplementary.pdf]

nanomaterials

Supplementary Materials

Efficient Removal of Cr(VI) Ions in Petrochemical Wastewater Using Fe_3O_4@*Saccharomyces cerevisiae* Magnetic Nanocomposite

Wei Long [1,2,*], Zhilong Chen [2], Jie Shi [2] and Shilin Yang [2]

[1] Guangdong Provincial Key Laboratory of Petrochemical Pollution Process and Control, Guangdong University of Petrochemical Technology, Maoming 525000, China
[2] College of Chemistry, Guangdong University of Petrochemical Technology, Maoming 525000, China
* Correspondence: longwei@gdupt.edu.cn; Tel.: +86-0668-2337639.

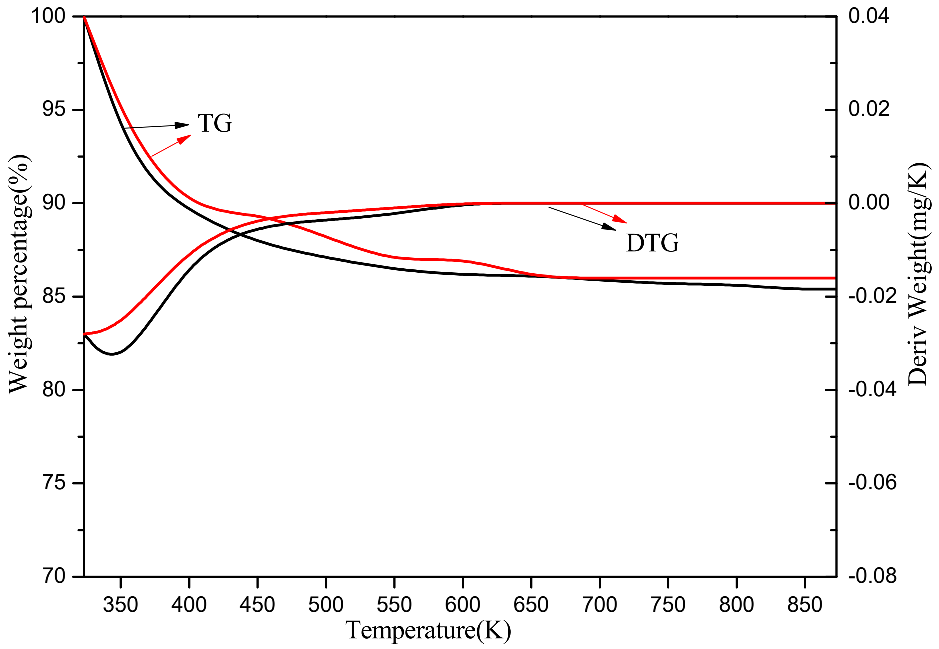

Figure S1. The TG and DTG analysize of 10%-Fe_3O_4@*SC* adsorbent. (Black: fresh. Red: after adsorption.)

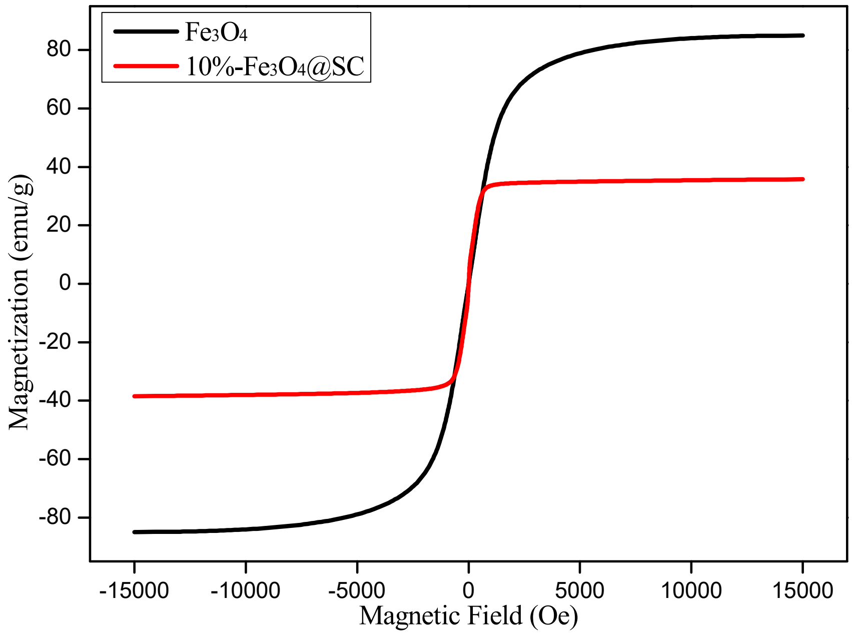

Figure S2. The VSM analysis of Fe_3O_4 and 10%-Fe_3O_4@SC sample. (Black: Fe_3O_4. Red: 10%-Fe_3O_4@SC sample.)

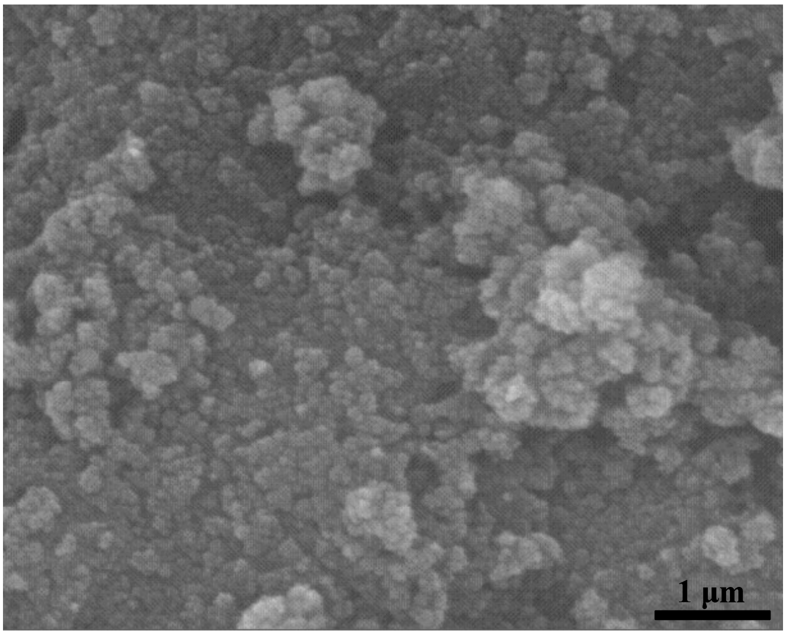

Figure S3. SEM image of *SC* adsorbent.

Table S1. Textural properties of *SC* and x%-Fe_3O_4@*SC* absorbents.

Absorbent	Surface area (m^2/g)	Average pore size (nm)	Pore volume (cm^3/g)	Average particle size (nm)
SC	182.91	17.9	0.68	30.8
3%-Fe_3O_4@SC	179.33	17.6	0.61	31.0
7%-Fe_3O_4@SC	180.71	17.3	0.60	32.3
10%-Fe_3O_4@SC	182.54	17.1	0.58	34.7
15%-Fe_3O_4@SC	157.42	14.4	0.51	41.9
20%-Fe_3O_4@SC	101.37	11.3	0.31	52.1

Note: Average diameter of particle size determined by the nitrogen adsorption-desorption.

Table S2. The adsorption performance by 10%-Fe_3O_4@*SC* adsorbent at lower pH.

pH	Adsorption time (h)	Equilibrium concentrations[a] (mg/L)	Equilibrium concentrations[b] (mg/L)	Adsorption capacity[a] (mg/g)	Adsorption capacity[b] (mg/g)
5.0	1	61.12	61.13	31.46	31.45
5.0	2	46.86	46.87	55.24	55.23
5.0	3	35.26	37.82	74.56	70.31
6.0	1	66.35	66.36	22.75	22.74
6.0	2	52.41	52.42	45.99	45.96
6.0	3	41.92	42.72	63.47	62.14
7.0	1	71.91	71.91	13.49	13.49
7.0	2	60.83	60.84	31.95	31.94
7.0	3	52.83	52.83	45.28	45.27

Adsorption conditions: the initial Cr(VI) ions concentration, 80 mg/L; volume, 50 mL; adsorbent mass, 0.03 g; temperature, 25°C; agitation speed, 400 rpm.

[a]The data were analyzed from the diphenylcarbazide method.

[b]The data were analyzed from atomic absorption spectrometry.

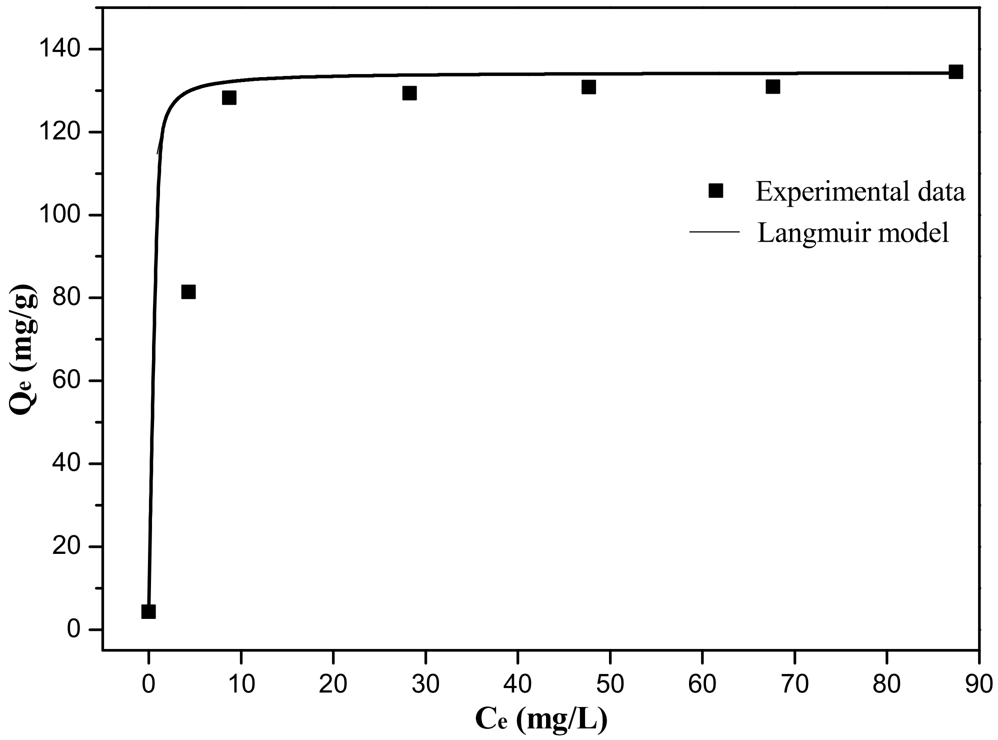

Figure S4. Test data fitting curve of Langmuir adsorption isotherm model.

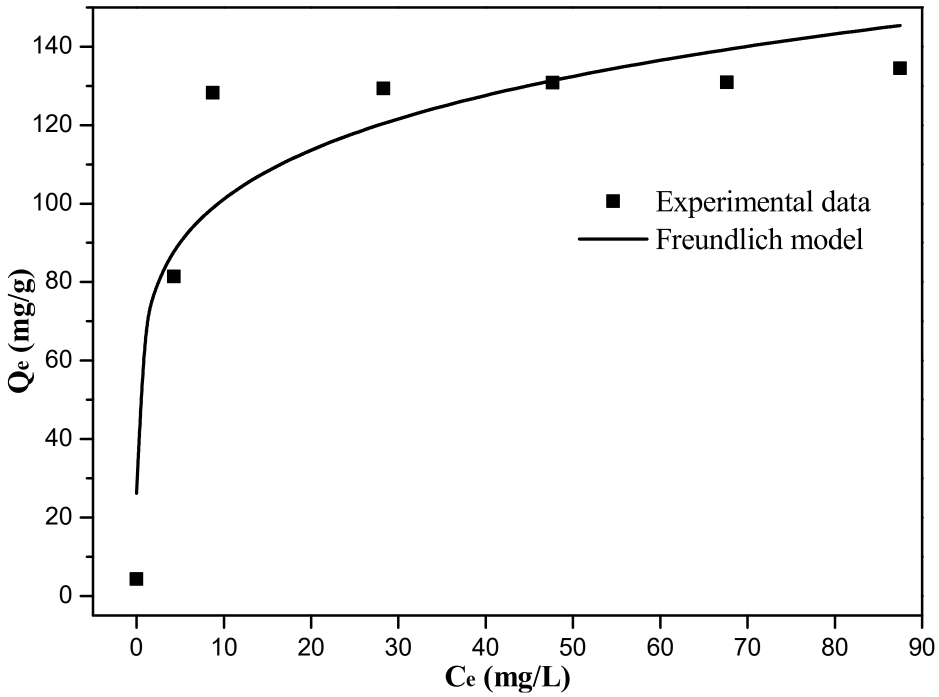

Figure S5. Test data fitting curve of Freundlich adsorption isotherm model.

Table S3. The experimental data of Langmuir isotherm at different temperatures.

288 K		298 K		308 K		318 K		328 K	
C_e (mg/L)	C_e/Q_e (g/L)	C_e (mg/L)	C_e/Q_e (g/L)	C_e (mg/L)	C_e/Q_e (g/L)	C_e (mg/L)	C_e/Q_e (g/L)	C_e (mg/L)	C_e/Q_e (g/L)
0.01	0.008	0.01	0.009	0.01	0.011	0.01	0.014	0.01	0.021
4.29	0.051	4.31	0.052	4.56	0.057	4.79	0.062	4.93	0.071
8.67	0.065	8.72	0.068	8.87	0.071	8.92	0.075	9.05	0.082
27.12	0.218	28.28	0.219	28.46	0.252	29.13	0.261	30.19	0.273
47.56	0.361	47.68	0.365	48.32	0.394	49.46	0.403	50.17	0.416
65.38	0.486	67.66	0.517	69.45	0.553	70.49	0.589	72.33	0.593
86.42	0.612	87.50	0.651	89.13	0.662	91.33	0.678	93.45	0.691

Table S4. The experimental data of Freundlich isotherm at different temperatures.

288 K		298 K		308 K		318 K		328 K	
$\ln C_e$	$\ln Q_e$	$\ln C_e$	$\ln Q_e$	$\ln C_e$	$\ln Q_e$	$\ln C_e$	$\ln Q_e$	$\ln C_e$	$\ln Q_e$
0.03	1.382	0.03	1.410	0.03	1.413	0.03	1.427	0.03	1.512
1.476	3.367	1.459	3.411	1.462	3.425	1.473	3.476	1.515	3.492
2.234	4.159	2.166	4.123	2.204	4.238	2.225	4.462	2.249	4.613
3.463	4.633	3.342	4.562	3.452	4.619	3.546	4.873	3.675	4.926
3.914	4.727	3.865	4.769	3.923	4.811	4.036	4.904	4.218	5.012
4.234	4.811	4.215	4.874	4.308	4.923	4.579	5.321	4.683	5.436
4.537	4.869	4.472	4.901	4.935	5.034	5.034	5.448	5.214	5.617

Table S5. Parameters of adsorption kinetic and intraparticle diffusion model.

**Models**	**Parameters**	**value**
Pseudo-first-order equation	$Q_{e\,(cal)}$ (mg/g)	138.99
	k_1 (min^{-1})	0.0182
	R^2	0.9911
Pseudo-second-order equation	$Q_{e(cal)}$ (mg/g)	201.61
	k_2 (g·mg^{-1}·min^{-1})	5.15×10^{-5}
	R^2	0.9517

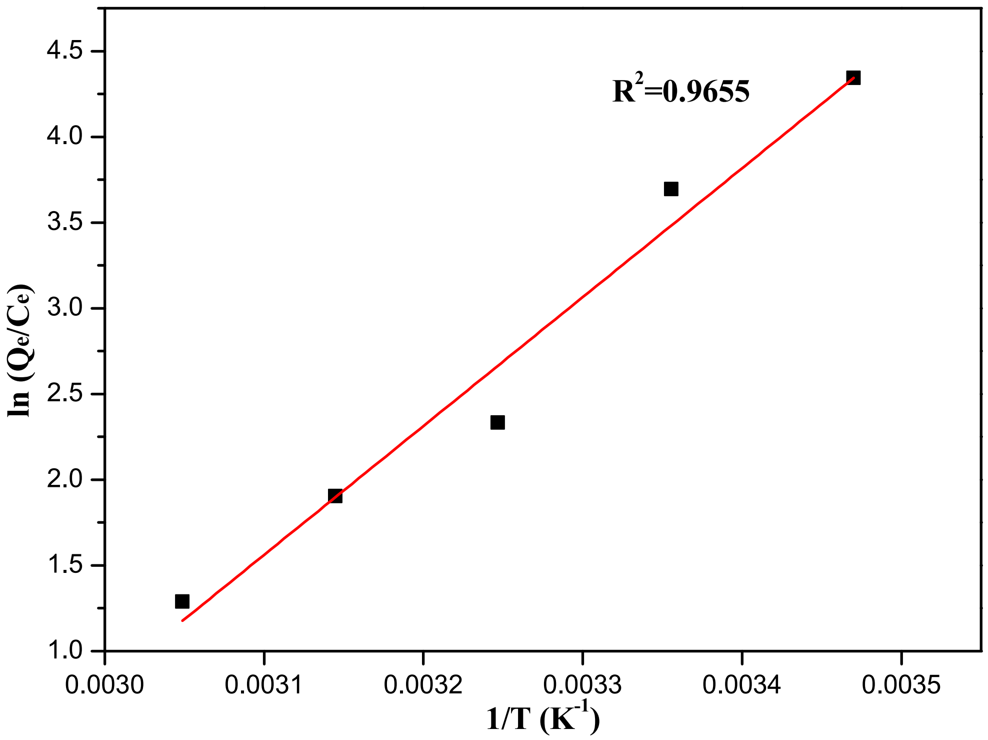

Figure S6. The plot dependence of ln(Q_e/C_e) on 1/T.

Table S6. The recyling adsorption performance by the *SC* adsorbent.

Cycle	Adsorption capacity(mg/g)	Removal rate of Cr(VI) ions(%)	Adsorption capacity*(mg/g)
1	100.42	75.32	97.49
2	70.42	52.82	70.46
3	66.32	49.74	66.39
4	54.19	40.64	54.34
5	42.25	31.69	41.19

Adsorption conditions: the initial Cr(VI) ions concentration, 80 mg/L; volume, 50 mL; pH, 2.0; adsorbent mass, 0.03 g; temperature, 25°C; adsorption time, 3 h; agitation speed, 400 rpm.
* The data were analyzed from atomic absorption spectrometry.
